# The clinical global impression scale and the influence of patient or staff perspective on outcome

**DOI:** 10.1186/1471-244X-11-83

**Published:** 2011-05-14

**Authors:** Thomas Forkmann, Anne Scherer, Maren Boecker, Markus Pawelzik, Ralf Jostes, Siegfried Gauggel

**Affiliations:** 1 Institute of Medical Psychology and Medical Sociology, University Hospital of RWTH Aachen, Aachen, Germany; 2 EOS Hospital for Psychotherapy, Münster, Germany

## Abstract

**Background:**

Since its first publication, the Clinical Global Impression Scale (CGI) has become one of the most widely used assessment instruments in psychiatry. Although some conflicting data has been presented, studies investigating the CGI's validity have only rarely been conducted so far. It is unclear whether the improvement index CGI-I or a difference score of the severity index CGI-S_ dif _is more valid in depicting clinical change. The current study examined the validity of these two measures and investigated whether therapists' CGI ratings correspond to the view the patients themselves have on their condition.

**Methods:**

Thirty-one inpatients of a German psychotherapeutic hospital suffering from a major depressive disorder (age M = 45.3, SD = 17.2; 58.1% women) participated. Patients filled in the Beck Depression Inventory (BDI). CGI-S and CGI-I were rated from three perspectives: the treating therapist (THER), the team of therapists involved in the patient's treatment (TEAM), and the patient (PAT). BDI and CGI-S were filled in at admission and discharge, CGI-I at discharge only. Data was analysed using effect sizes, Spearman's *ρ *and intra-class correlations (ICC).

**Results:**

Effect sizes between CGI-I and CGI-S _dif _ratings were large for all three perspectives with substantially higher change scores on CGI-I than on CGI-S _dif_. BDI_ dif _correlated moderately with PAT ratings, but did not correlate significantly with TEAM or THER ratings. Congruence between CGI-ratings from the three perspectives was low for CGI-S _dif _(ICC = .37; Confidence Interval [CI] .15 to .59; *F*_30,60 _= 2.77, *p *< .001; mean *ρ *= 0.36) and moderate for CGI-I (ICC = .65 (CI .47 to .80; *F*_30,60 _= 6.61, *p *< .001; mean *ρ *= 0.59).

**Conclusions:**

Results do not suggest a definite recommendation for whether CGI-I or CGI-S _dif _should be used since no strong evidence for the validity of neither of them could be found. As congruence between CGI ratings from patients' and staff's perspective was not convincing it cannot be assumed that CGI THER or TEAM ratings fully represent the view of the patient on the severity of his impairment. Thus, we advocate for the incorporation of multiple self- and clinician-reported scales into the design of clinical trials in addition to CGI in order to gain further insight into CGI's relation to the patients' perspective.

## Background

The Clinical Global Impression Scale (CGI) is a brief clinician-rated instrument that consists of three different global measures. 1. *Severity of illness: *overall assessment of the current severity of the patient's symptoms (CGI-S); 2. *Global improvement: *overall comparison of the patient's baseline condition with his current state (CGI-I); 3. *Efficacy index: *overall comparison of the patient's baseline condition to a ratio of current therapeutic benefit and severity of side effects (CGI-E). Since its first publication the CGI has become one of the most widely used assessment tools in psychiatry [1]. For example, the CGI, especially the CGI improvement scale (CGI-I) has been widely utilized as an efficacy measure in clinical drug trials in different mental disorders [e.g., depression, schizophrenia; [2,3]]. Its popularity is mainly based on its conciseness and easiness of administration.

It is widely accepted and some studies presented evidence arguing that the CGI is a valid assessment instrument. Moreover, the CGI was used as external criterion to test the validity of other outcome measures such as the Beck Depression Inventory [BDI; [4]], the Hamilton Depression Rating Scale [HAMD; [5]] or the Montgomery-Asberg Depression Rating Scale [MADRS; [6-9]].

Despite its general acceptance and extensive use as outcome measure and criterion for the validation of other instruments, the CGI's psychometric characteristics have only rarely been examined so far. Some evidence has been presented arguing for its validity when used in clinical trials [10]. Beyond that, in a recent meta-analysis, Hedges et al. [11] calculated effect sizes for CGI and other rating scales from 16 different studies on social phobia and found mostly comparable effect sizes for the CGI-I and several social anxiety scales. In line with that, Khan et al. [12] found similar effect sizes for MADRS, HAMD and CGI in antidepressant clinical trials which were interpreted by the authors as supporting the CGI's sensitivity.

However, from early on, the CGI has been criticized for being inconsistent, unreliable and too general to measure clinical conditions or treatment responses validly [13,14]. Guy [15] draws attention on the role of memory when using the CGI-I and claimed that the task to compare a patient's general clinical condition at study end to that at the beginning of the study using the CGI is essentially a test of the rater's memory. Recently, more empirical evidence for this criticism has been presented. Busner et al. [16] found that the CGI ratings of the clinicians are affected by indication-irrelevant adverse events reported by the patient. Participants were asked to rate the severity of a major depressive disorder or a generalized anxiety disorder and nausea or dizziness served as indication irrelevant medical events. The more such events being reported by the patient, the more likely the clinician rated the patient as more severely ill. The authors concluded that these reports can threaten validity of the CGI seriously. Jiang and Ahmed [17] found evidence for relatively low correlation between CGI-S and CGI-I which raised the question of whether it is more appropriate to use the CGI-I or a difference between CGI-S pre and CGI-S post intervention to judge change across treatment.

A couple of different efforts have been made to improve the psychometric characteristics of the CGI. Kadouri et al. [18] tested the use of a semi-structured interview, a new response format and a Delphi process to improve reliability of the CGI. Best results were found when ratings of four different clinical raters were averaged. Targum et al. [19] found significantly augmented scoring variance due to treatment emergent symptoms and developed targeted scoring criteria for the CGI to enhance inter-rater reliability. Another attempt to improve the CGI's psychometric quality was the development of alternative versions of the CGI for use in special patient groups [e.g., [20]].

To sum up, results of studies on the psychometric performance of the CGI are mixed. Additional research appears necessary. More precisely, the question of whether the CGI provides a valid measure of the patient's condition and if so whether it is more appropriate to use CGI-I or a difference score of CGI-S as outcome criterion is not ultimately answered. The current study therefore addressed this issue. First, we aimed at clarifying whether the CGI provides a valid measure of the patient's condition. For this purpose, it was investigated whether CGI ratings correspond to the view the patient has on his or her current condition. If so, clinician rated CGI scores should relate to patient rated CGI scores and scores on other patient reported outcome measures. Furthermore - in correspondence with findings from Kadouri et al. [18] - we expected that this relation improves if not a single clinician does the rating but a whole team of therapists using a consensus process. Second, starting from the results of Jiang and Ahmed [17] this study assessed whether it is valid to rely on the CGI-I when rating clinical change or whether calculating difference scores for CGI-S at the beginning and the end of the intervention would enhance validity. Based on Guy's [15] notion on the role of memory when using CGI-I we expected that difference scores for CGI-S were the more valid measure. Implications for clinical practice will be discussed.

## Methods

### Sample

The sample consisted of 31 inpatients of a German psychotherapeutic hospital suffering from a major depressive disorder (MDD) according to the criteria of the 10^ th ^edition of the International Classification of Diseases (ICD-10). Diagnoses were verified in a two step procedure: First, depression was assessed by the treating therapist using a clinical interview in which the International Diagnostic Checklist for depression (IDCL) [21] was applied. The IDCL is a checklist that can be used to make a careful evaluation of the symptoms and classification criteria, and thus help to arrive at precise diagnoses according to ICD-10 criteria for a depressive episode. If the therapist was still unsure about the diagnosis after using IDCL, the German Version of the Structured Clinical Interview for DSM-IV (SKID) [22] was conducted in addition. The clinical interviews were conducted by clinical psychologists. In the second step, diagnoses were verified through clinical conferences including senior psychotherapists and psychiatrists. Mean age of patients was 45.3 years (SD = 17.2), 58.1% were women. Patients stayed at hospital for 41 days (SD = 28.4) on average. Since it was a convenience sample, it reflected all "facets", levels, and stages of chronicity of depression. All participants took part voluntarily without payment and signed an informed consent prior to testing. The study procedures were in accordance with the declaration of Helsinki and approved by the local ethics committee of the Medical Faculty of the RWTH Aachen University (EK 172/05). See table 1 for sample details.

**Table 1:** Sample details

	M	SD
Age	45.3	17.2

	**N**	**%**

Gender (female)	18	58.1
Comorbidity		
F1x.xx	4	12.9
F4x.xx	10	32.3
F5x.xx	4	12.9
F6x.xx	7	22.6
F9x.xx	1	3.2
No comorbidity	5	16.1

At the hospital, patients are treated on an inpatient basis with high-density empirically-based psychotherapy that is personalized depending on the disorder of the patient. The program uses symptom-focused and highly individualized interventions. Each inpatient is treated by only one therapist for as long as eight hours per day.

High-density psychotherapy typically includes four phases: (1) Psychological assessment and a medical examination from which feedback is given to the patient, as is information about the therapy program. This phase includes 6-8 sessions, and it lasts one or two days. (2) Cognitive preparation for therapy is given to enhance the patient's motivation for specific treatment exercises. The patient's core assumptions about the aetiology of his or her disorder are taken into account when the treatment plan is devised. The therapist explains to the patient the details of the therapy and the subsequent steps to be taken. (3) During this phase, specific therapeutic exercises are carried out. These include standard elements of cognitive behavioural therapy for depression. (4) The self-management phase begins after several days of high-density psychotherapy. At the beginning of this phase, the therapist helps the patient to plan and organize the tasks to be undertaken; thereafter, the patient is asked to independently devise difficult tasks to do. Finally, the difficulties that the patient has in completing the tasks are evaluated. After discharge, therapists remain in telephone contact with their patients for at least six weeks.

### Material

#### Beck Depression Inventory (BDI)

The BDI contains 21 items [4]. Each item consists of four self-referring statements (e.g. "I am sad"). Item scores range from 0 to 3 and participants are supposed to choose one or more statements per item that represents best their mental state during the last week. A total score >10 indicates mild to moderate depression and a total score >18 moderate to severe depression. The BDI was filled in at admission and discharge.

#### Clinical Global Impression Scale (CGI)

The CGI consists of three global measures. The CGI severity of illness measure (CGI-S) is rated from 1 (normal, not at all ill) to 7 (among the most extremely ill patients). A "0" is allocated if the patient was not assessed. The CGI-S was rated at admission (CGI-S_adm_) and at discharge (CGI-S_ dis_). The CGI global improvement measure (CGI-I) is rated from 1 (very much improved) to 7 (very much worse). Again, "0" stands for "not assessed". The CGI-I was rated at discharge only. The third measure is called the efficacy index CGI-E. It was not assessed in the current study [1].

The CGI measures were rated from three perspectives: the treating therapist (THER), the team of therapists concerned with the patient (TEAM), and the patient him- or herself (PAT). The team of therapists concerned with the patient performed a delphi process to reach a consensus rating of the respective patient's condition.

### Data analysis

#### CGI-I vs. CGI-S _dif_

Difference scores for CGI-S (CGI-S _dif _= CGI-S_adm_-CGI-S_ dis_) were determined and contrasted to CGI-I ratings for all three perspectives to determine congruence of the two global ratings. Additionally, effect sizes *d *between CGI-S _dif _and CGI-I and their confidence intervals (95%) were calculated for all three perspectives. If the confidence interval for the ES includes zero, the effect can be regarded as statistically nonsignificant. In order to reduce sampling error effect sizes have been corrected using a factor provided by Hedges and Olkin [23]. Following Cohen [24] effect sizes .20 <*d *≤ .50 were interpreted as small, .50 <*d *≤ .80 as medium, and *d *≥ .80 as large. Before calculating effect sizes, CGI-S _dif _was rescaled for this step of analysis into values from 1 to 7 with 4 meaning no change in order to bring CGI-I and CGI-S_ dif _to a common metric. Above, both CGI-I and CGI-S _dif _were correlated (Spearman's *ρ*) with BDI difference scores (BDI_ dif _= BDI_adm_-BDI _dis_).

#### Congruence between patients', therapists' and teams' perspectives on CGI-S and CGI-I

Means and standard deviations (SD) for CGI-S_adm_, CGI-S _dis _and for CGI-I were calculated. Corrected effect sizes *d *were calculated between CGI-S_adm _and CGI-S _dis _for all three perspectives. Afterwards, measures of congruency between the three perspectives were calculated. Because interval scale level of data collected with the CGI could not be taken for granted we decided to report both measures for interval scale level data and measures for ordinal scale level data. As measures of congruency for interval scale level data intraclass correlations (ICC) according to McGraw and Wong [25] were calculated separately for CGI-S_adm_, CGI-S_ dis_, and CGI-I to determine congruency of the patients', therapists' and team's ratings on these three global measures. In addition, Spearman's *ρ *for ordinal scale level data was determined. Significance level was set at α = .05.

All analyses were conducted using SPSS 17 for Windows.

## Results

### CGI-I vs. CGI-S_ dif_

**Figure 1:**
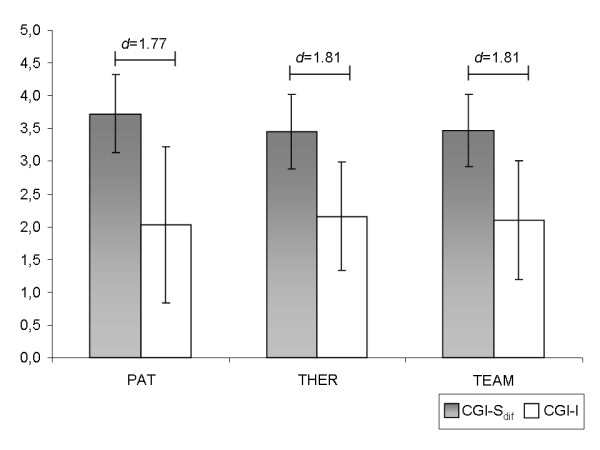
**Effect sizes between CGI-I and CGI-S_dif _for the perspectives patient (PAT), therapist (THER) and team of therapists concerned with the patient (TEAM)**. Higher scores indicate greater change between admission and discharge.

On average, patients, therapists and teams rated the patient's condition on CGI-I with a "2" indicating "much improvement" [1] (see table 2). By contrast, the rescaled difference values between CGI-S_adm _and CGI-S _dis _revealed an averaged improvement of 3.55 (SD = 0.57). A value of "4" indicates no change. Effect sizes between CGI-I and CGI-S_ dif _ratings were large for all three perspectives (see figure 1) with substantially higher change scores on CGI-I than on CGI-S_ dif_. Correlations (Spearman's *ρ*) with BDI_ dif _were *ρ*_BDIdif/CGI-I-PAT  _= -.39 (p = .02), *ρ*_BDIdif/CGI-I-THER  _= -.16 (p = .34), *ρ*_BDIdif/CGI-I-TEAM  _= -.23 (p = .16), *ρ*_BDIdif/CGI-S-dif-PAT _ = .29 (p = .07), *ρ*_BDIdif/CGI-S-dif-THER  _= .24 (p = .13) and *ρ*_BDIdif/CGI-S-dif-TEAM  _= -.08 (p = .60). Thus, results suggest that BDI_dif _correlated moderately with ratings from the patients' perspective, but did not correlate significantly with ratings from the therapists' or teams' perspective. Correlations with BDI_ dif _thus differed between perspectives but not between CGI-I and CGI-S_ dif_.

**Table 2:** Mean ratings on CGI-I and CGI-S at admission and discharge from all three perspectives

	admission		discharge		difference			effect size	
	**M**	**SD**	**M**	**SD**	**M**	**SD**	***d***	**Lower CI**	**Upper CI**

CGI-S patient	4.00	1.90	3.45	1.50	.55	1.18	0.32	-0.18	0.82
CGI-S therapist	4.97	0.71	3.87	1.09	1.10	1.14	1.18	0.64	1.72
CGI-S team	5.00	0.63	3.94	0.77	1.10	1.09	1.48	0.92	2.05
BDI	20.24	8.41	10.68	7.88	9.56	9.09	1.15	0.56	1.75
CGI-I patient	--	--	2.03	1.20	--	--	--	--	--
CGI-I therapist	--	--	2.16	.82	--	--	--	--	--
CGI-I team	--	--	2.10	.91	--	--	--	--	--

Effect sizes of the differences between admission and discharge are corrected according to Hedges and Olkin [21].

### Congruence between patients', therapists' and teams' perspectives on CGI-S and CGI-I

Mean CGI-S_adm _ratings at admission were 4.0 (SD = 1.9) for the patient, 4.97 (SD = 0.71) for the therapist, and 5.0 (SD = 0.63) for the team perspective. At discharge all mean ratings dropped: patients' CGI-S_ dis _mean ratings were 3.45 (SD = 1.50), therapists' were 3.87 (SD = 1.09), and teams' ratings were 3.94 (SD = 0.77). The resulting effect sizes *d *differed substantially (*d*_Patient _= .32; *d*_therapist _= 1.18; *d*_team _= 1.48). The effect size for the patient perspective was markedly smaller than for the other two perspectives which coincided with a much bigger standard deviation. Effect size for BDI sum scores was large (d_BDI _= 1.15; M_adm _= 20.2, SD_adm _= 8.4; M_dis _= 10.7, SD_dis _= 7.9).

CGI-S_adm _(ICC = .22; Confidence Interval [CI] .00 to .46; *F*_30,60 _= 1.82, *p *= .02; mean *ρ *= 0.29) and CGI-S_ dis _(ICC = .24; CI .03 to .48; *F *_30,60 _= 1.97, *p *= .01; mean *ρ *= 0.59) ratings as well as the differences CGI-S_ dif _between both (ICC = .37; CI .15 to .59; *F*_30,60 _= 2.77, *p *< .001; mean *ρ *= 0.36) showed low ICCs indicating low congruency of ratings between the three perspectives. In all three cases, the ratings from the patient's perspective showed substantially lower intercorrelations with the ratings from the other two perspectives (see table 3).

**Table 3:** Intercorrelations between the three perspectives for CGI-I, CGI-S_adm_, CGI-S_dis_, and CGI-S_dif_

		CGI-I				CGI-S_adm_				CGI-S_dis_				CGI-S_dif_		
		**patient**	**therapist**	**team**		**patient**	**therapist**	**team**		**patient**	**therapist**	**team**		**patient**	**therapist**	**team**

**Patient**	**CGI-I**	1.00			**CGI-S adm**	1.00			**CGI-S dis**	1.00			**CGI-S dif**	1.00		
**therapist**		*0.51*	1.00			0.30	1.00			0.27	1.00			0.29	1.00	
**team**		*0.53*	*0.73*	1.00		0.20	*0.78*	1.00		0.08	*0.54*	1.00		0.20	*0.58*	1.00

**M**	0.42				0.29				0.59				0.36			

*Note. *M = mean; correlations in italics are significant at *α *= .05.

Mean CGI-I ratings were 2.03 (SD = 1.20) for the patient, 2.16 (SD = .82) for the therapist and 2.10 (SD = .91) for the team perspective. The intraclasscorrelation between the patients', therapists' and team's ratings on CGI-I was ICC = .65 (CI .47 to .80; *F*_30,60 _= 6.61, *p *< .001; mean *ρ *= 0.42) indicating moderate to high agreement between the ratings from the three perspectives.

## Discussion

The current study aimed at investigating the validity of the CGI-I and CGI-S_ dif _as outcome measures in clinical trials. More precisely, it was examined whether use of CGI-I or CGI-S_ dif _appears more appropriate. Above, it was investigated whether therapists' CGI ratings correspond to the view the patients themselves have on their condition.

The results of the present study showed that CGI-I provided relatively high change scores compared to the difference score CGI-S_ dif _in terms of effect sizes. To rate a patient's condition on the CGI-I clinicians first have to remember the patient's condition at admission and then contrast it to their condition at present. By contrast, CGI-S only needs representation of the patient's current condition. Thus, the current results might be interpreted as suggesting that using CGI-I might be more prone to well known effects of hindsight memory distortion [e.g., [26]]: When using CGI-I at discharge, therapists, teams and patients might have been inclined to retrospectively recall the patient's condition at admission as more impaired than it really was according to CGI-S_adm _and thus rated change of condition as more prominent. If this was the case, in our view, it would threaten the validity of CGI-I as outcome measure in clinical trials. However, additional research is needed directly addressing the role of memory effects on results in CGI-I until a definite conclusion on this issue is possible.

The congruence of ratings from the three perspectives on CGI-I was moderate to good and much better than the congruence of ratings on CGI-S. Moreover, while congruence between the single therapists and the teams was moderate to good, patients gave divergent ratings especially on CGI-S _dif_. Overall, patients provided the most conservative ratings for change, in both CGI-I and CGI-S_ dif_. Simultaneously, patients' ratings correlated most strongly with BDI_ dif _for both CGI-I and CGI-S_ dif _while correlations with BDI for the other two perspectives were virtually zero. One might oppose that doubts on the validity of a self-reported CGI-rating might be warrantable because originally the CGI was not designated to be a self-rated scale so that low correlations with self-reported CGI could be seen as weak criterion for validity. However, self-reported CGI-ratings correlated significantly with BDI and the validity of BDI as an instrument for the assessment of depression severity has been shown in numerous studies [for some recent examples see e.g., [27,28]]. These results suggest that CGI ratings - regardless of whether CGI-I or CGI-S _dif _are concerned - made by the treating therapist or obtained through a consensus process in the team of therapists appear not to fully represent the view of the patient on the severity of his or her impairment.

So which global measure of CGI should be used as outcome measure, CGI-I or CGI-S _dif_? Results of the present study do not suggest a definite recommendation since no strong evidence for the validity of neither CGI-I nor CGI-S_ dif _could be found. In our view, the overall picture of results could be interpreted as being slightly in favour for CGI-I but without doubt additional research is needed.

As already noted, there were no substantial differences between therapists' and teams' ratings. One potential explanation is that in our study the therapist who did the single rating was also member of the team of therapists and might have influenced the consensus rating in his favoured direction. Nevertheless, at least under the conditions described, our results suggest that in contrast to Kadouri et al. [18] a consensus rating following a Delphi process does not necessarily change reliability or validity of the rating.

A couple of limitations of the current study have to be reported. The sample size was rather small so that reported results should be interpreted with care. Above, only patients suffering from a MDD have been assessed which impedes generalizability of the reported results to other patient groups. Because the length of the current depressive episode could not be determined from study data, it could not be ruled out that length of depressive episode or chronicity could have had an influence on results. Furthermore, since neither the CGI nor the BDI have been applied to a random sample of the adult population the rather low to moderate ICC found in the present study might simply be explained by the fact that only a very homogeneous sample consisting of patients who had been hospitalized for MDD has been investigated. Replication studies, ideally with larger and more heterogeneous samples are warranted.

The only criterion available for the validation of the CGI in this study was self-reported data (BDI and patients' ratings on CGI). However, the most valid procedure for diagnosing a depressive disorder is a structured diagnostic interview based on DSM-IV [29] or ICD-10 [30] criteria that is conducted by a clinical expert. Thus, future studies should incorporate interview-based assessments at discharge for replication of the present findings.

The reported findings were not collected in a clinical trial which is one of the main areas of application for CGI. In clinical trials clinicians are usually blinded as to what study condition the patient belongs, e.g., treatment vs. placebo. Thus, they do not know whether it is supportive for the aim of the study to state that the patient improved much or not. However, in this study, clinicians treated *and *rated the patients themselves. It might therefore be possible that clinicians might have been inclined to assign relatively high change scores. However, they also knew that the conducted study did *not *aim at evaluating therapy effects so that we expect the effect of such demand characteristics in our data to be rather small. Nevertheless, future research should investigate whether our results could be replicated in a blinded setting.

## Conclusions

In summary, in line with previous research [16,17,19] the results of the present study cast doubt on the validity of the CGI. To our knowledge, this is the first study that included correspondence of clinician rated CGI scores with the patients' own perspective on their clinical condition as one criterion of validity. Our results do not suggest a definite recommendation for whether CGI-I or CGI-S_ dif _should be used since no strong evidence for the validity of neither CGI-I nor CGI-S_ dif _in terms of high correlations with ratings from the patients' perspective could be found. We conclude that it cannot be recommended to rely upon CGI alone as outcome measure in clinical trials but rather advocate for the incorporation of multiple self- and clinician-reported scales into the design of clinical trials in addition to CGI in order to gain further insight into CGI's relation to the patients' perspective.

## Competing interests

The authors declare that they have no competing interests.

## Authors' contributions

TF contributed to conception and design of the study, conducted the statistical analysis and wrote the manuscript. AS participated in the analysis and interpretation of the data. MB participated in the design of the study and the statistical analysis. RJ and MP participated in the design of the study and coordinated the data acquisition. SG has been involved in drafting and revising the manuscript, and coordinated the study and data acquisition. All authors read and approved the final manuscript.

## Pre-publication history

The pre-publication history for this paper can be accessed here:

http://www.biomedcentral.com/1471-244X/11/83/prepub
